# *In-utero* exposure to tenofovir disoproxil fumarate pre-exposure prophylaxis and growth metrics in HIV unexposed breastfed infants in South Africa: a *post hoc* analysis of the CAP 016 PrEP in pregnancy RCT

**DOI:** 10.3389/fped.2024.1447173

**Published:** 2024-08-09

**Authors:** Megeshinee Naidoo, Kimesh L. Naidoo, Carl Lombard, Alicia C. Desmond, Richard Clark, James F. Rooney, Glenda Gray, Dhayendre Moodley

**Affiliations:** ^1^ Centre for the Program of AIDS Research in South Africa (CAPRISA), Durban, South Africa; ^2^ Department of Paediatrics and Child Health, School of Clinical Medicine, University of KwaZulu Natal, Durban, South Africa; ^3^ Biostatistics Unit, South African Medical Research Council, Tygerberg, South Africa; ^4^ Division of Epidemiology and Biostatistics, Department of Global Health, University of Stellenbosch, Tygerberg, South Africa; ^5^ Gilead Sciences Inc, Foster City, CA, United States; ^6^ South African Medical Research Council, Cape Town, South Africa; ^7^ Department of Obstetrics and Gynaecology, School of Clinical Medicine, University of KwaZulu Natal, Durban, South Africa

**Keywords:** *in-utero*, exposure, preexposure prophylaxis (PrEP), growth, breastfeeding

## Abstract

**Objective:**

We evaluated growth metrics in HIV unexposed African breastfed infants in the first 18 months of life in association with *in-utero* exposure to Tenofovir Diphosphate Fumarate (TDF) containing pre-exposure prophylaxis (PrEP).

**Design:**

We conducted a secondary data analysis of a TDF-PrEP randomized control trial (CAP016 RCT). Pregnant women without HIV were randomized to initiating TDF-PrEP in pregnancy (Immediate-PrEP-IP) or deferred initiation of TDF-PrEP at cessation of breastfeeding (Deferred-PrEP-DP).

**Methods:**

Infant weight (W), length (L), and head circumference (HC) were measured at birth and 6, 26, 50, and 74 weeks of age. Stored dried blood spot samples from pregnant women randomized to the IP arm were used to measure tenofovir-diphosphate (TFV-DP) levels. Age-stratified mean weight-for-age (WAZ), length-for-age (LAZ), weight-for-length (WLZ), and head circumference-for-age (HCAZ) *Z*-scores were compared between infants exposed to varying TFV-DP concentrations and infants in the DP arm.

**Results:**

A total of 455 mother-infant pairs were included in the secondary analysis, 228 in the IP arm and 227 in the DP arm. WAZ, LAZ, WLZ, and HCAZ scores were comparable between infants in the Deferred-PrEP arm and Immediate-PrEP arm. In a mixed-effects linear regression model adjusting for maternal age, body mass index, socioeconomic and newborn characteristics, *in-utero* exposure to varying TFV-DP levels was not associated with WAZ (*β* = −0.52), LAZ (*β* = −0.46), WLZ (*β* = −0.43) and HCAZ (*β* = −0.11) scores over time.

**Conclusion:**

There was no evidence of an association between growth metrics in the first 18 months of life and *in-utero* exposure to TFV-DP among breastfed HIV unexposed infants.

## Introduction

Oral pre-exposure prophylaxis (PrEP), most commonly available as a combination of Tenofovir Disoproxil Fumarate (TDF) and Emtricitabine-triphosphate (FTC), is widely used by pregnant and lactating women since the World Health Organisation (WHO) launched its recommendations in 2017 (1–4). Tenofovir (TFV) is known to readily cross the placenta with cord blood concentrations reaching 70%–100% of maternal plasma concentration (5). While several studies have provided reassuring evidence that *in-utero* exposure to TDF does not affect pregnancy and newborn outcomes (6–8), a systematic review of adult studies concluded that TDF was associated with lower bone mineral density in adults taking TDF as part of a combination treatment or as PrEP (9). A similar trend was reported for infants who were born to women living with HIV and receiving TDF-containing antiretroviral treatment (10, 11). Consequentially, growth trajectories in HIV-exposed and uninfected infants have been well described, however, studies determining the association between *in-utero* exposure to TDF-containing antiretroviral treatment and growth have yielded inconsistent findings (12–16).

As more children become exposed to maternal TDF-based PrEP in sub-Saharan Africa, it remains important to understand how this might impact the growth of children already predisposed to nutritional and developmental challenges. Growth outcomes in HIV-unexposed infants and those exposed to *in-utero* TDF-based PrEP are limited (17–20). Since poor adherence to PrEP use during pregnancy and postpartum period is reported as a common challenge in PrEP implementation studies (21–23), we aimed to determine if there was an association between growth metrics and *in-utero* exposure to TDF-PrEP as measured by tenofovir diphosphate (TFV-DP) concentration in red blood cells using dried blood spots collected during pregnancy.

## Methods

The CAP 016 clinical trial was an open-label randomized control study designed to investigate the safety of TDF/FTC when used as oral PrEP in healthy, HIV-uninfected pregnant women at substantially high risk for HIV infection (7). Pregnant women meeting eligibility criteria and providing informed consent were randomized in a 1:1 ratio to immediate initiation of PrEP in pregnancy (Immediate PrEP Arm) or deferred initiation of PrEP at the cessation of breastfeeding (Deferred PrEP Arm) using a computer-generated permuted block (block size of ten) randomisation list and sealed envelopes with sequential participant study numbers. Gestational age was determined in all participants using ultrasound scanning at the enrolment visit before 28 weeks. Following randomization, women were seen every four weeks until delivery. For women randomized to the Immediate PrEP Arm, the pharmacist dispensed a monthly supply of once-daily oral Truvada (TDF 300 mg and FTC 200 mg). Participants delivered at the regional hospital near the research clinic. A delivery visit was conducted within seven days of delivery at which, labour and delivery data were extracted from the maternity chart and mothers and newborns were examined by the study clinician. All women and infants were given monthly appointments for the first three months after delivery and thereafter three-monthly appointments until 74 weeks. Women remained on their assigned treatment arm until breastfeeding cessation. Data included in this *post hoc* analysis was collected between September 2017 and December 2020.

As a *post hoc* investigation, TFV-DP was measured in red blood cells using stored dried blood spots (DBS) that were collected at two visits during pregnancy. Extractions from a 50-*μ*l DBS were tested for TFV-DP (fmol/3-mm punch) using high-performance liquid chromatography–tandem mass spectrometry at the University of Cape Town, South Africa. The lower limit of quantification for tenofovir diphosphate was 16.6 fmol/3-mm punch. Extrapolating benchmark adherence metrics from the study by Stranix-Chibanda et al, which estimated the TNF-DP concentration of 93 fmol/punch to correspond to 1 dose/week (24). We used a threshold of 500 fmol/punch which equates to five doses/week representing 70% adherence to the daily oral TDF-PrEP regimen.

At birth, the infant was assessed and the mother's intention to breastfeed was documented. Women were encouraged to breastfeed their infants for as long as possible. Infants were assessed if brought in by the mother at 6, 26, 50, and 74 weeks of age. To ensure optimum retention, these visits coincided with routine infant immunization visits. At each visit, a trained research assistant measured the infant weight, length, and head circumference using a calibrated infant weighing scale, a stadiometer, and a non-stretchable measuring tape respectively. The World Health Organisation (WHO) growth standards were used to calculate age and sex-appropriate *Z*-scores for weight, length, and head circumference (25). Infant feeding modality was recorded at each visit. Cessation of breastfeeding was defined as 28 days after the last exposure to breast milk.

Maternal sociodemographic characteristics included age, level of education, and monthly household income. Infant birth characteristics included infant sex, gestational age at birth, weight, length, head circumference, infant feeding practice, duration of breastfeeding, and weight-for-age, length-for-age, weight-for-age and head circumference-for-age *Z*-scores.

Mother-infant pairs were included in this analysis if infants were assessed at more than one scheduled visit at 6, 26, 50, and 74 weeks. The maternal TFV-DP levels were categorized as <500 and >500 fmol/punch. Maternal sociodemographic characteristics and infant birth characteristics were compared between TDF-unexposed infants and infants exposed to maternal TFV-DP level <500 fmol/punch and >500 fmol/punch using *t*-tests for numeric data and Chi-square tests or Fisher's exact test (small frequencies) for categorical data. Student *t*-tests assuming unequal variances were used to compare WAZ, LAZ, WLZ, and HCAZ scores at 6, 26, 50, and 74 weeks between TDF-unexposed, maternal TFV-DP level <500 fmol/punch and >500 fmol/punch groups. For the adjusted analysis a mixed linear regression model was used to compare the growth trajectories between groups over time while adjusting for maternal socioeconomic status, age, low birth weight, and infant sex. A correlation coefficient (*β*) with 95% confidence intervals was reported for WAZ, LAZ, WLZ and HCAZ scores in association with no exposure to TDF-PrEP and *in-utero* exposure to TFV-DP <500 and >500 fmol/punch. A two-sided *P*-value of less than 0.05 indicated significance for the primary analyses. SAS 9.4 (NC, USA) was used for all analyses.

Ethical approval of the CAP 016 study was granted by the Institutional Review Board of the University of KwaZulu-Natal (UKZN IRB), BFC 243/16, for which all participants signed a written informed consent. Written informed consent for the original study was obtained before any study procedures were performed and included consent for infant participation following delivery as well as consent for data collected to be used in future research studies.

## Results

**Figure 1:**
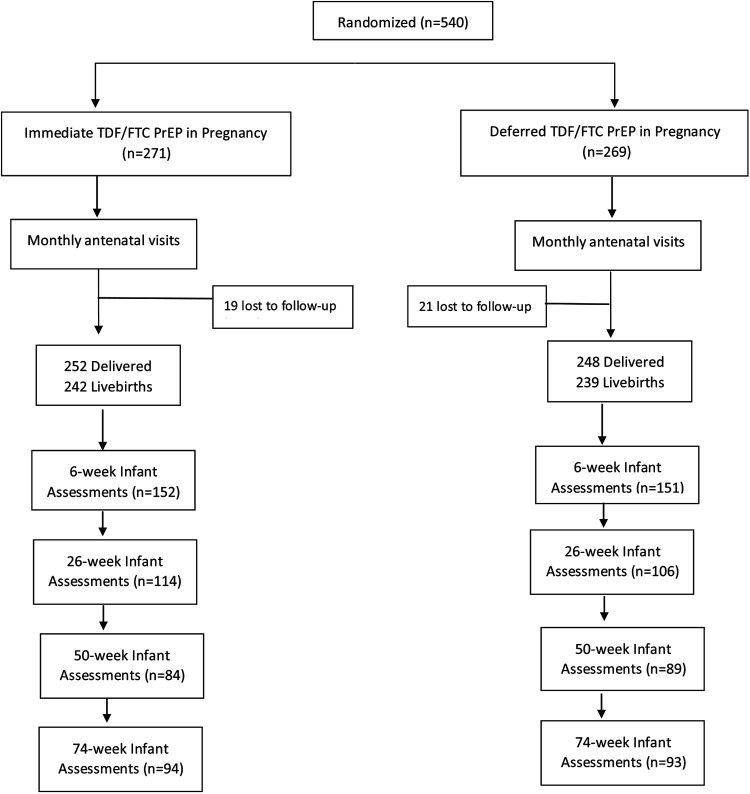
Consort diagram of Infant Assessments by Randomisation Arm.

A total of 455 mother-infant pairs were included in the secondary analysis, 228 in the Immediate PrEP arm and 227 in the Deferred PrEP arm. Overall, 303 infants were assessed at 6 weeks, 220 infants at 26 weeks, 173 infants at 50 weeks, and 187 infants at 74 weeks (Figure 1). Among 228 women randomized to the Immediate PrEP arm, the mean TFV-DP level during pregnancy was 310.3 fmol/punch ranging from undetectable to 946.7 fmol/punch. Among the 228 women, 99 (43.4%) women had TFV-DP levels ranging from undetectable to 200 fmol/punch indicating one or less dose/week (<15% adherence); 61 (26.8%) women had TFV-DP levels between 200 and 500 fmol/punch (two to 4 doses/week) and 68 with >500 fmol/punch (five or more doses/week). Overall, we compared the growth metrics of 68 infants exposed to TFV-DP levels >500 fmol/punch with 160 infants exposed to less than 500 fmol/punch and 227 infants in the Deferred PrEP arm.

The median age (IQR) of women included in this secondary analysis was 23 (20; 27) with 62% between 18 and 25 years of age. More than half of the women (61.8%) lived in low-income households reporting a monthly income of less than R1200. Baseline sociodemographic characteristics were comparable between women randomized to the deferred PrEP arm, and women randomized to the Immediate PrEP arm with TFV-DP <500 fmol/punch and TFV-DP >500 fmol/punch (Table 1). 91.4% of infants were born at term (>37 weeks) and 92% of normal birth weight (>2,500 g). Birth anthropometric measures were comparable between groups except for the lowest mean HCAZ score (0.11) reported for infants born to women in the TFV-DP >500 fmol/punch group. The vast majority (78.5%) of infants had initiated breastfeeding, however, the mean duration of breastfeeding was relatively short (23 weeks) with the majority (64.6%) breastfed for less than 6 months.

**Table 1:** Characteristics of women at enrolment and infants by *in-utero* exposure to TDF-PrEP.

	Deferred PrEP Arm *N* = 227	TFV-DP < 500 fmol/punch *N* = 160	TFV-DP > 500 fmol/punch *N* = 68	*P*-value	TOTAL *N* = 455
Maternal					
Age, years [median (IQR)]	23 (20;26)	23 (21;27)	23 (20.5; 27)	0.361	23 (20; 27)
Age category *n* (%)					
18 ≤ 24 years	143 (63.0)	97 (60.6)	43 (63.2)		283 (62.2)
>25 years	84 (37.0)	63 (39.4)	25 (36.8)	0.878	172 (37.8)
Level of education *n* (%)					
Primary or secondary school	53 (23.4)	35 (21.9)	12 (17.7)		100 (22.0)
Completed matric (grade 12)	174 (76.7)	125 (78.1)	56 (82.4)	0.609	355 (78.0)
Household income *n* (%)					
<R1200	146 (64.3)	94 (58.8)	41 (60.3)		281 (61.8)
≥R1200	81 (35.7)	66 (41.2)	27 (39.7)	0.521	174 (38.2)
Infant					
Sex *n* (%)					
Male	123 (54.2)	80 (50.0)	37 (54.4)		240 (52.8)
Female	104 (45.8)	80 (50.0)	31 (45.6)	0.688	215 (47.2)
Infant gestation					
Mean (SD) weeks	38.9 (1.9)	39.0 (1.8)	38.6 (1.5)	0.201	38.9 (1.8)
Preterm <37 weeks *n* (%)	22 (9.7)	11 (6.9)	6 (8.8)		39 (8.6)
Term ≥37 weeks *n* (%)	205 (90.3)	149 (93.1)	62 (91.2)	0.619	416 (91.4)
Birth weight					
Mean (SD)	3.1 (0.5)	3.2 (0.5)	3.1 (0.5)	0.079	3.1 (0.5)
WAZ mean (SD)	−0.49 (1.08)	−0.26 (1.19)	−0.52 (1.01)	0.094	−0.42 (1.13)
Low birth weight *n* (%)	16 (7.1)	13 (8.2)	7 (10.3)		36 (8.0)
Normal birth weight *n* (%)	210 (92.9)	145 (91.8)	61 (89.7)	0.679	416 (92.0)
Birth length, cm					
Mean (SD)	49.5 (2.3)	49.8 (2.6)	49.2 (2.4)	0.256	49.5 (2.4)
LAZ mean (SD)	−0.03 (1.19)	0.13 (1.38)	−0.18 (1.28)	0.209	0.003 (1.29)
Birth head circumference, cm					
Mean (SD)	34.6 (1.4)	34.9 (1.5)	34.3 (1.3)	0.019	34.6 (1.5)
HCAZ mean (SD)	0.29 (1.19)	0.57 (1.23)	0.11 (1.12)	0.016	0.36 (1.21)
Infant feeding practices *n* (%)					
Ever breastfed	174 (76.7)	129 (80.6)	54 (79.4)		357 (78.5)
Never breastfed	53 (23.4)	31 (19.4)	14 (20.6)	0.632	98 (21.5)
Breastfeeding duration					
Mean (SD), in weeks	24.9 (27.4)	20.4 (25.6)	24.5 (25.3)	0.239	23.2 (26.5)
Duration < 6 months *n* (%)	136 (59.9)	115 (71.9)	43 (63.2)		294 (64.6)
Duration ≥ 6 months *n* (%)	91 (40.1)	45 (28.1)	25 (36.8)	0.051	161 (35.4)

Age-stratified mean WAZ, LAZ, WLZ, and HCAZ scores were comparable between infants born to women randomized to the Deferred PrEP arm and infants exposed to maternal TFV-DP <500 and >500 fmol/punch (Table 2). Mean LAZ scores were consistently low across all groups from 6 weeks, PrEP Unexposed (−1.06), TFV-DP <500 (−0.89) and TFV-DP > 500 (−1.12) through to 74 weeks, PrEP Unexposed (−0.82), TFV-DP <500 (−0.76) and TFV-DP > 500 (−1.03) (Table 2).

**Table 2:** Age stratified growth metrics in PrEP unexposed infants vs. Exposed to *in-utero* TFV-DP Concentration <500 fmol/punch and >500 fmol/punch.

Study visit	Outcome	Deferred PrEP Arm *N* = 227		TFV-DP < 500 fmol/punch *N* = 160		TFV-DP > 500 fmol/punch *N* = 68		*P*-value
		*N*	Mean (SD)	*N*	Mean (SD)	*N*	Mean (SD)	
6 weeks	WAZ	151	0.02 (1.20)	103	0.35 (1.22)	49	0.03 (1.17)	0.079
	LAZ	151	−1.06 (1.41)	103	−0.89 (1.33)	49	−1.12 (1.27)	0.519
	WLZ	151	1.48 (1.40)	102	1.73 (1.41)	49	1.55 (1.39)	0.359
	HCAZ	151	0.02 (1.68)	103	0.27 (1.65)	49	0.05 (1.63)	0.485
26 weeks	WAZ	106	0.66 (131)	75	1.02 (1.03)	39	0.66 (1.20)	0.116
	LAZ	106	−0.56 (1.51)	74	−0.12 (1.17)	39	−0.53 (1.28)	0.086
	WLZ	106	1.43 (1.43)	74	1.51 (1.05)	39	1.39 (1.20)	0.714
	HCAZ	105	0.78 (1.60)	75	1.24 (1.39)	39	1.19 (1.22)	0.082
50 weeks	WAZ	89	0.87 (1.33)	55	1.20 (1.12)	29	0.64 (1.23)	0.113
	LAZ	89	−0.66 (1.50)	55	−0.21 (1.22)	29	−0.59 (1.39)	0.166
	WLZ	89	1.59 (1.29)	55	1.74 (1.15)	29	1.23 (0.95)	0.185
	HCAZ	89	1.23 (1.46)	55	1.60 (1.32)	29	1.14 (1.24)	0.222
74 weeks	WAZ	93	0.89 (1.55)	69	1.39 (1.18)	25	0.59 (1.63)	0.510
	LAZ	93	−0.82 (1.69)	68	−0.76 (1.50)	25	−1.03 (1.93)	0.776
	WLZ	93	1.69 (1.61)	68	1.81 (1.21)	25	1.46 (1.26)	0.501
	HCAZ	93	1.31 (1.44)	69	1.48 (1.46)	25	1.11 (1.52)	0.525

WAZ, weight-for-age *z*-score; LAZ, length-for-age *z*-score; WLZ, weight for-length *z*-score; HCAZ, head circumference-for-age *z*-score.

In a mixed-effects linear regression model adjusting for maternal age, BMI, socioeconomic and newborn characteristics, *in-utero* exposure to high TFV-DP levels (>500 fmol/punch) was not associated with WAZ [*β*=−0.52 (95%CI −1.10 to 0.06)], LAZ [*β*=−0.46 (95%CI −1.10 to 0.18)], WLZ [*β* = −0.43 (95%CI −1.05 to 0.18)] and HCAZ [*β* = −0.11 (−0.76 to 0.55) scores over time (Table 3).

**Table 3:** *In-utero* exposure to TFV-DP concentration as predictors for WAZ, LAZ, WLZ and HCAZ scores in HIV unexposed infants.

	Linear regression model *β* (95%CI)	*P*-value	Multivariable Linear mixed-effects Regression Model^a^ β (95%CI)	*P*-value
Weight-for-age *Z*-score				
TFV < 500 fmol/punch	0.06 (−0.39 to 0.51)	0.797	−0.24 (−0.79 to 0.31)	0.390
TFV > 500 fmol/punch	−0.35 (−0.83 to 0.14)	0.160	−0.52 (−1.10 to 0.06)	0.077
Length-for-age *Z*-score				
TFV < 500 fmol/punch	0.17 (−0.34 to 0.68)	0.512	−0.30 (−0.92 to 0.31)	0.335
TFV > 500 fmol/punch	−0.17 (−0.71 to 0.37)	0.535	−0.46 (−1.10 to 0.18)	0.160
Weight-for-length *Z*-score				
TFV < 500 fmol/punch	−0.20 (−0.66 to 0.26)	0.383	−0.26 (−0.85 to 0.32)	0.381
TFV > 500 fmol/punch	−0.38 (−0.87 to 0.11)	0.127	−0.43 (−1.05 to 0.18)	0.164
Head circumference-for-age *Z*-score				
TFV < 500 fmol/punch	0.06 (−0.47 to 0.59)	0.821	0.14 (−0.49 to 0.77)	0.655
TFV > 500 fmol/punch	−0.16 (−0.72 to 0.40)	0.577	−0.11 (−0.76 to 0.55)	0.746

WAZ, weight-for-age *z*-score; LAZ, length-for-age *z*-score; WLZ, weight for-length *z*-score; HCAZ, head circumference-for-age *z*-score.

^a^ adjusted for maternal age, BMI, socioeconomic characteristics and newborn characteristics.

**Figure 2:**
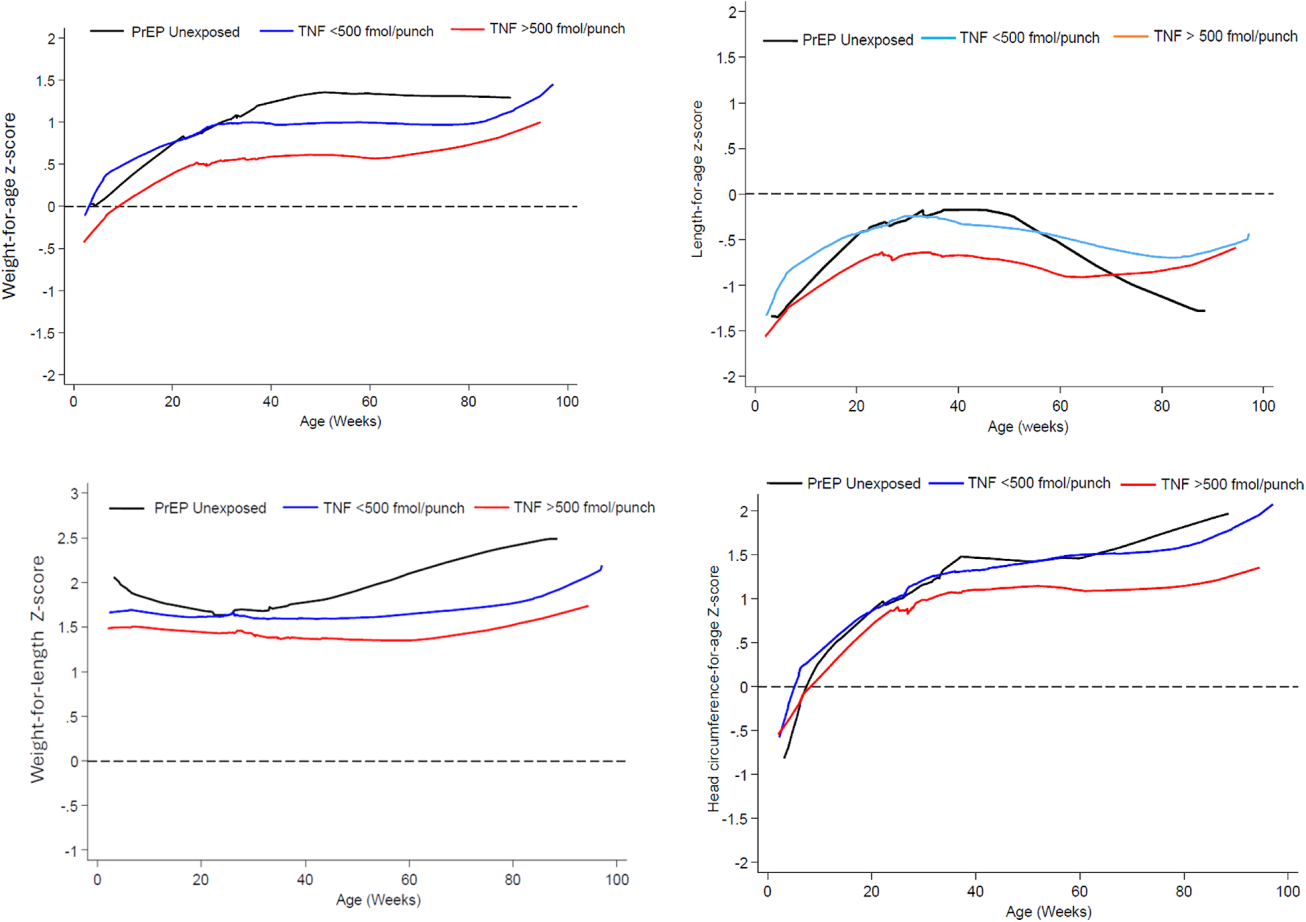
Mean weight-for-age, length-for-age, weight-for-length and head circumference-for-age Z-score from 6 t0 74 weeks by prEP exposure and *in-utero* TFV-DP concentration exposure.

Between 6 and 50 weeks, mean LAZ, WAZ, WLZ and HCAZ scores were lowest with *in-utero* exposure of TFV-DP >500 fmol/punch while there was much overlap in the LAZ, WAZ and WLZ profiles for the PrEP unexposed and TNF-DP <500 fmol/punch exposed infants in the first 26 weeks of life (Figure 2). Beyond 50 weeks, while other growth metrics showed an upward trend, only the mean LAZ score decreased for the PrEP unexposed group to levels below that of the TFV exposed groups.

## Discussion

In a *post hoc* analysis of a randomized non-inferiority PrEP in pregnancy clinical trial, *in-utero* exposure to high concentrations of TFV-DP, as opposed to lower TFV-DP concentrations or no-exposure to TDF-PrEP, was not associated with adverse growth trajectories in HIV-unexposed breastfed infants in the first 18 months of life. WAZ, LAZ, WLZ and HCAZ scores were lowest among infants exposed to high concentration of TFV-DP but not significantly lower than the other study groups.

Suboptimal adherence to daily oral PrEP in pregnancy and postpartum as demonstrated through TFV-DP concentrations in maternal blood has been previously reported (22, 23) and our study confirms the suboptimal adherence using an objective assessment. Our findings of the lack of an association between growth measures and *in-utero* TDF-based PrEP exposure are consistent with other TDF-based PrEP safety studies, although few studies explored the effect of high *in-utero* exposure to TFVDP on growth anthropometrics. Although studies such as the PrEP Implementation for Mothers in Antenatal Care (PrIMA) intend to report TFV-DP levels as an objective method of assessing adherence, recently released growth monitoring beyond 12 months did not include findings that were dependent on an objective assessment of adherence (17). At 24 months, there was no difference in mean weight, mean height, frequency of underweight, and stunting between children with and without prenatal PrEP exposure (18). Growth measures remained comparable at 30 months. In the Partners Demonstration Project, in sensitivity analysis restricted to infants whose mothers had tenofovir detected there was no evidence that 17 infants born to women with detectable TFV-DP levels had poorer growth profiles by 12 months of age when compared to the 49 PrEP unexposed infants (19). In the PrEP Implementation in Young Women and Adolescents (PrIYA) program that delivered PrEP to pregnant and postpartum women integrated within routine maternal and child health clinics in Kenya, early growth monitoring revealed that WAZ, WLZ and LAZ scores did not differ between PrEP exposed and unexposed infants at 6 weeks of age (20). This is probably one of the larger cohorts (206 PrEP exposed and 1,324 PrEP unexposed), but the short time frame for growth monitoring and the lack of an objective assessment of true *in-utero* exposure to TFV-DP renders these findings inconclusive.

Mean LAZ scores in our study were consistently lower than zero at all visits between 6 weeks and 18 months but not associated with TDF exposure. While the LAZ scores were lowest for the high-TFV-DP exposed group, it appeared to normalize after 12 months of age. On the other hand, infants not exposed to PrEP had higher LAZ scores in the first year of life and subsequently with decreased scores between 12 and 18 months of life. Lower LAZ scores and a higher prevalence of stunting among HIV exposed uninfected (HEU) infants in comparison to HIV unexposed uninfected (HUU) infants have previously been reported (13). These studies have implicated the shorter breastfeeding duration among HEU infants in poor linear growth. In our study, HUU infants in the non-PrEP arm were breastfed for longer however none had breastfed for longer than 12 months and cessation of breastfeeding before 12 months may be related to decreased linear growth after 12 months.

WAZ scores were lowest for infants exposed to higher TFV-DP concentrations and unlike other growth metrics in our study that improved over time, the WAZ score remained lower throughout the study follow-up period until 18 months. Further follow-up of growth parameters in infants beyond 18 months would be essential and the PrIMA-x study could provide valuable data after considering true *in-utero* TFV-DP exposure (18). Our study was not without limitations. The retention rate was low with many missed follow-up visits and early study terminations often seen as a result of internal migration common amongst women in the postpartum period. Furthermore, the study follow-up period overlapped with the COVID-19 pandemic affecting all clinical trial-related activities and operations. As such there were far fewer infants than anticipated that contributed to the data analysis.

## Conclusion

Although our findings are consistent with other PrEP studies, we provide additional evidence of a lack of association between growth restriction and *in-utero* TDF exposure using an objective assessment of PrEP exposure. While the small sample size makes interpretation of these results difficult, limiting its generalisability to other settings, it nevertheless provides important reassuring safety data on growth outcomes of infants exposed to maternal TDF-based PrEP.

## Data Availability

The raw data supporting the conclusions of this article will be made available by the authors, without undue reservation.
