# Supplementary material for: In-utero exposure to tenofovir disoproxil fumarate pre-exposure prophylaxis and growth metrics in HIV unexposed breastfed infants in South Africa: a post hoc analysis of the CAP 016 PrEP in pregnancy RCT
Source: Front Pediatr. 2024 Aug 9;12:1447173. doi: 10.3389/fped.2024.1447173 (PMC11341386; doi:10.3389/fped.2024.1447173)
Supplement: Supplementary file 1 [file Table1.pdf]

Supplementary Table 1: Maternal TNF-DP Dose effect on Infant Growth Outcomes between Birth and 18 months of age

**WAZ mean (SD)**	**n**	**BLQ**	**n**	**0_200**	**n**	**200_400**	**n**	**400_600**	**n**	**>600**	**p value**
Birth	35	-0.32 (1.15)	62	-0.37 (1.04)	41	0.05 (1.32)	48	-0.35 (1.21)	40	-0.69 (0.98)	0.051
6 wks	21	0.19 (1.30)	43	0.43 (1.21)	23	0.62 (1.02)	35	0.004 (1.50)	30	0.01 (0.78)	0.077
6 mths	16	1.24 (1.15)	25	0.87 (1.09)	22	1.27 (0.88)	27	0.47 (1.03)	24	0.82 (1.22)	0.055
12 mths	10	1.31 (1.57)	19	1.26 (0.92)	17	1.39 (1.16)	22	0.43 (0.90)	16	0.91 (1.36)	0.075
18 mths	12	1.25 (1.14)	28	1.07 (1.28)	16	0.87 (0.81)	23	0.51 (1.43)	15	0.86 (1.74)	0.384
**LAZ mean (SD)**											
Birth	36	0.14 (1.30)	60	0.08 (1.12)	41	0.42 (1.66)	48	-0.14 (1.39)	39	-0.30 (1.30)	0.052
6 wks	21	-1.12 (1.62)	43	-0.79 (1.22)	23	-0.55 (0.93)	35	-1.17 (1.54)	30	-1.18 (1.14)	0.114
6 mths	15	0.15 (1.18)	25	-0.34 (1.24)	22	0.22 (0.96)	27	-0.74 (1.22)	24	-0.32 (1.27)	0.042
12 mths	10	-0.30 (1.47)	19	-0.08 (1.15)	17	0.02 (1.13)	22	-0.96 (1.16)	16	-0.22 (1.49)	0.117
18 mths	12	-1.04 (1.54)	28	-0.81 (1.37)	16	-0.38 (1.18)	22	-1.05 (2.01)	15	-0.86 (1.98)	0.437
**WLZ mean (SD)**											
Birth	34	-0.64 (1.31)	59	-0.69 (1.47)	40	-0.46 (2.31)	45	-0.40 (1.67)	38	-0.79 (1.64)	0.758
6 wks	21	1.87 (1.21)	43	1.69 (1.64)	23	1.66 (1.28)	34	1.56 (1.37)	30	1.65 (1.35)	0.969
6 mths	15	1.52 (0.83)	25	1.53 (1.22)	22	1.59 (0.96)	27	1.31 (0.98)	24	1.94 (1.39)	0.731
12 mths	10	1.94 (1.36)	19	1.73 (0.95)	17	1.84 (1.43)	22	1.20 (0.79)	16	1.35 (1.04)	0.276
18 mths	12	2.29 (0.98)	28	1.92 (1.42)	16	1.41 (0.80)	22	1.37 (1.09)	15	1.70 (1.42)	0.167
**HCAZ mean (SD)**											
Birth	36	0.39 (0.95)	59	0.49 (1.17)	41	0.91 (1.17)	48	0.39 (1.41)	39	-0.06 (1.16)	0.011
6 wks	21	0.15 (1.99)	43	0.25 (1.55)	23	0.79 (1.36)	35	0.28 (1.87)	30	-0.40 (1.31)	0.132
6 mths	16	1.60 (1.38)	25	0.65 (1.44)	22	1.69 (1.14)	27	1.06 (1.30)	24	1.32 (1.25)	0.057
12 mths	10	1.22 (1.09)	19	1.64 (1.42)	17	1.76 (1.44)	22	1.14 (1.45)	16	1.40 (0.90)	0.704
18 mths	12	1.59 (1.39)	28	1.46 (1.65)	16	1.57 (1.08)	23	1.39 (1.79)	15	0.84 (0.95)	0.474

BLQ=below level of quantification, 0-200 fmol/punch, 200-400 fmol/punch, 400-600 fmol/punch and >600 fmol/punch

WAZ=Weight-for-age z-score, LAZ=Length-for-age z-score, WLZ=weight-for-length z-score, HCAZ=Head circumference-for-age z-score
